# Prognosis of lymphadenectomy in malignant ovarian germ cell tumor

**DOI:** 10.3389/fonc.2023.1143893

**Published:** 2023-04-17

**Authors:** Bin Lv, Xinghui Liu, Ai Zheng, Ling Han

**Affiliations:** ^1^ Department of Obstetrics and Gynecology, West China Second University Hospital, Sichuan University, Chengdu, Sichuan, China; ^2^ Key Laboratory of Birth Defects and Related Diseases of Women and Children (Sichuan University), Ministry of Education, Sichuan, China

**Keywords:** lymphadenectomy, malignant ovarian germ cell tumor, ovarian cancer, survival rate, recurrence

## Abstract

**Background and objectives:**

The routine application of lymphadenectomy remains a controversial part of surgical staging in malignant ovarian germ-cell tumor (MOGCT). Thus, studies are needed to explore the prognostic significance of lymphadenectomy in MOGCT. The goal of this retrospective study was to report the clinical outcomes of lymph node dissection (LND) and non-LND in MOGCT surgeries.

**Measurements and main results:**

A total of 340 cases of MOGCTs were included: 143 patients (42.1%) had LND and 197 (57.9%) had no LND. The OS rates at 5 years in the LND and non-LND group were 99.3% vs. 100%, respectively. The DFS rates at 5 years in the LND and non-LND group were 88.8% vs. 88.3%. Forty-three patients (12.6%) were successfully pregnant during the postoperative follow-up. There were 44 recurrences (12.9%) and six deaths (1.8%). Stage was an independent prognostic factor for DFS in the multivariate analysis. Pathology was reported as an independent prognostic factor associated with OS in the multivariate analysis.

**Conclusion:**

Lymphadenectomy had no significant influence on the OS (P=0.621) or disease-free survival rate (P=0.332) of patients with MOGCT.

## 1 Introduction

Malignant ovarian germ-cell tumor (MOGCT) is a rare ovarian cancer that accounts for approximately 2–3% % of all ovarian cancer (1) and usually occurs in young women. It is unilaterally diagnosed at an early stage, and characterized by rapid growth, high malignancy, and high chemosensitivity (2). With the development of adjuvant chemotherapy for MOGCT, the remission rate has increased. Comprehensive surgical staging combined with chemotherapy is the standard treatment method for MOGCT, except for stage IA dysgerminoma and stage IA grade I immature teratoma. Conservative surgery with preservation of the uterus and contralateral ovary is preferred for young women who wish to preserve fertility.

Routine application of lymphadenectomy is a controversial part of surgical staging in MOGCT. Lymphadenectomy can remove potentially metastatic lesions and identify the FIGO stage, which can help guide postoperative treatment. However, lymphadenectomy is associated with serious intraoperative and postoperative complications, such as blood vessel injury and lymphedema. Previous studies have shown differences in the prognositic effect of lymphadenectomy in MOGCT. Several studies have shown that lymphadenectomy did not improve patient survival in the early stages of MOGCT (3–5), while others have reported that lymphadenectomy is an independent predictor of survival and associated with a higher risk of disease recurrence in the early stage (6, 7). Thus, further studies are needed to explore the prognostic significance of lymphadenectomy in MOGCT. The goal of this study was to report the clinical outcomes of LND and non-LND in MOGCT surgeries.

## 2 Materials and methods

This retrospective, observational, single-center study was conducted at a tertiary hospital in Chengdu, China. A cohort of women diagnosed with MOGCT between January 1, 2007, and January 1, 2017, were included. The study was approved by the Ethics Committee of the West China Second University Hospital, Sichuan University (2021-M-185).

According to the final pathological diagnosis, the tumor stage was determined according to the International Federation of Gynecology and Obstetrics (FIGO) 2014 classification. Patients staged before 2014 were restaged according to the 2014 International Federation of Gynecology and Obstetrics criteria. The inclusion criteria were: 1) patients diagnosed with MOGCT, 2) known surgical treatment, known age, 3) known histology type, and 4) willingness to participate in follow-up at a time of ≥ 5 years. The exclusion criteria were: unknown lymph node dissection status, age, histologic type, and extent of surgical treatment.

In total, 379 cases of MOGCT were collected; 340 cases were included, and 39 cases were excluded due to incomplete data. The following detailed basic information of the included patients was also recorded. The following basic information: patient’s age, tumor size, histopathology, and FIGO stage. The following perioperative information was also recorded: the surgical route, operation time, blood loss, perioperative complications, number of lymph node metastases, omentectomy, hysterectomy, and adjuvant chemotherapy status. Further details of the oncological and pregnancy outcome were also recorded: the number of patients with recurrence, who died, and who were pregnant.

Patients were divided into two groups according to the surgical information collected: the LND and non-LND group. Patients who underwent systemic pelvic and/or para-aortic lymphadenectomy were classified into the lymphadenectomy group; 143 patients (42.1%) had LND and 197 (57.9%) had no lymph node dissection. Survival duration was defined as the period from surgery until date of recurrence (disease-free survival [DFS]) and death (overall survival [OS]). The number of dissected lymph nodes was calculated based on the pathology report. The rate of lymph node metastasis was calculated based on the number of patients with positive lymph nodes and who underwent lymphadenectomy. SPSS version 25.0 (IBM, Armonk, NY, USA) was used to conduct all statistical analyses. The demographic and clinical characteristics were compared between the LND and non-LND group. All continuous variables in this study were normally distributed, thus they were analyzed using the t-test, and are presented as mean ± standard deviation. Categorical variables are presented as frequency and percentage, and the χ2 test or Fisher’s exact test was used, depending on which was appropriate. Propensity score matching was used to avoid selection bias caused by the demographic and clinical characteristics between the LND and non-LND group. These characteristics included age, tumor size, histopathology, FIGO stage, surgery route, omentectomy, hysterectomy, and adjuvant chemotherapy. A propensity score analysis with one-to-one matching using a caliper of 0.02 was conducted. Survival curves were constructed using the Kaplan–Meier method with a log-rank test. Cox proportional hazards models were used to conduct survival analysis. Hazard ratios (HRs) and their 95% confidence intervals (CI) were calculated for multivariate analysis using characteristics with *P*<0.2 in univariate analysis. A *P* value less than 0.05 was considered statistically significant.

## 3 Results

**Figure 1:**
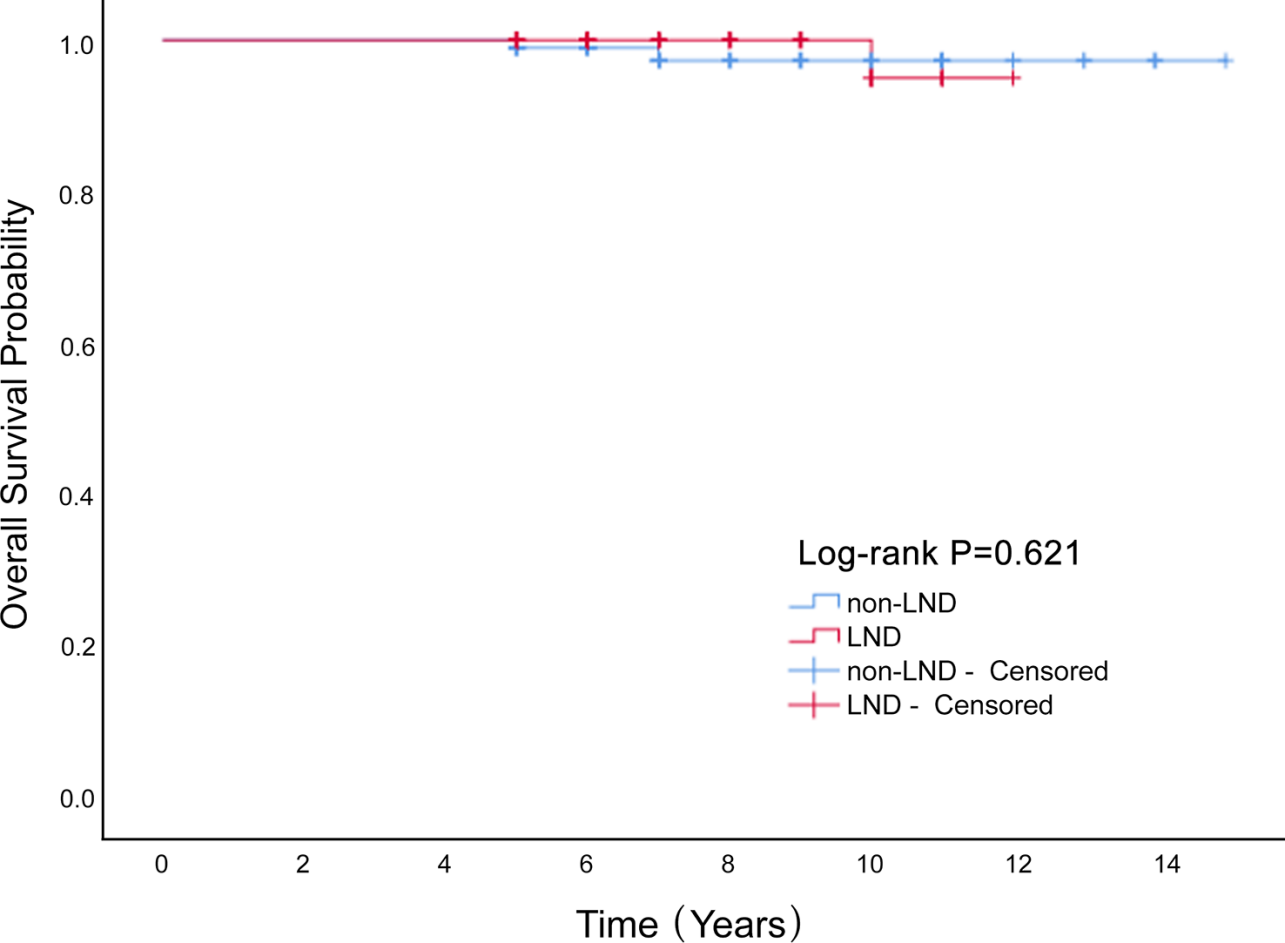
The Overall Survival rate of LND and non-LND groups of MOGCT patients.

**Figure 2:**
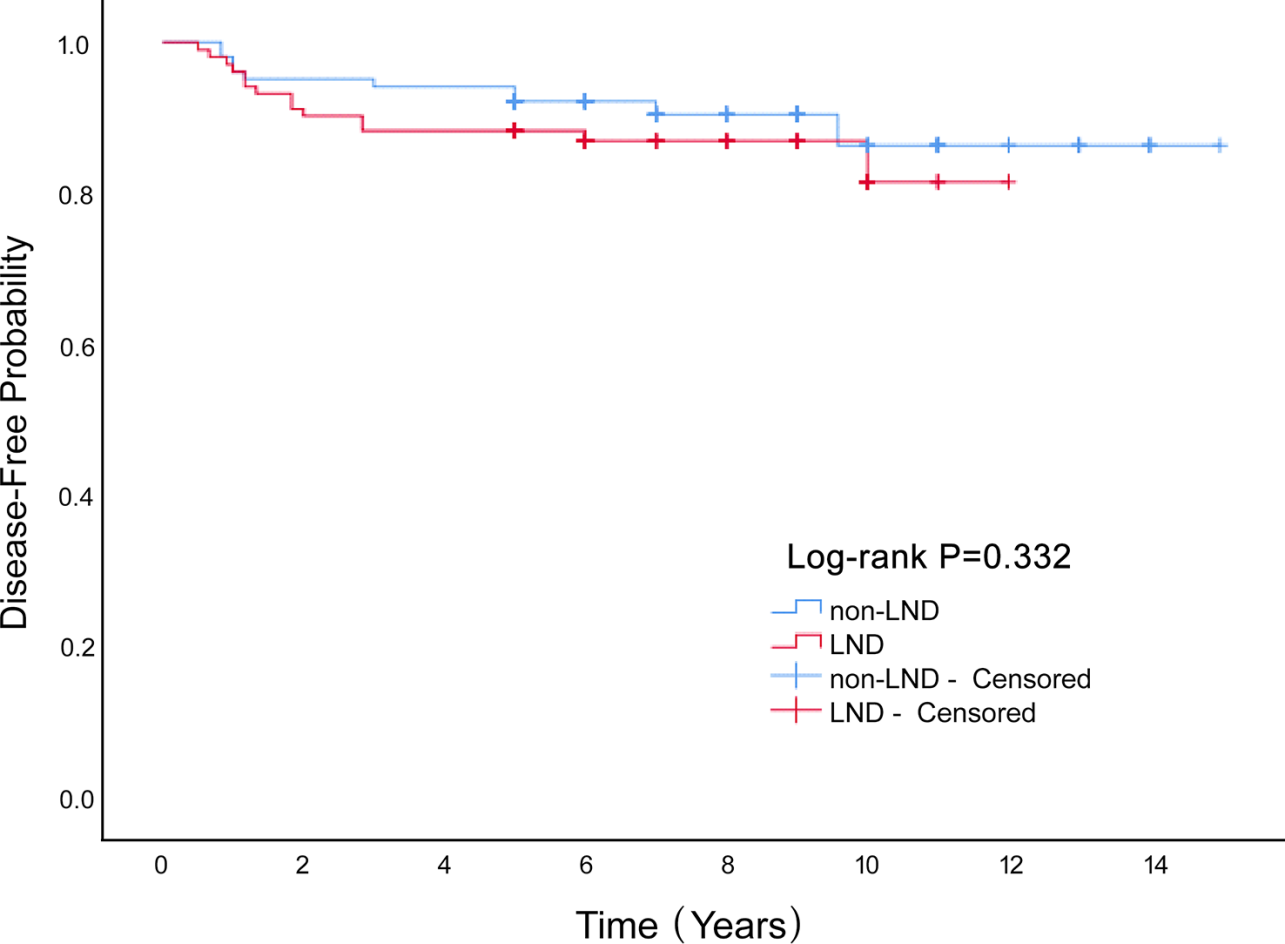
The Disease-Free survival rate of LND and non-LND groups of MOGCT patients.

We enrolled 340 patients who underwent surgery, and were fully followed-up for five years or more. LND cases constituted 42% of the entire cohort. The average age was 25.3 ± 11.2 years, and the average diameter of the tumor was 12.7 ± 6.3 cm. These included immature teratoma, 128 (37.6%); dysgerminoma, 64 (18.8%); yolk-sac tumor, 109 (32.1%); and mixed germ-cell tumor, 39 (11.5%). In our study, 43 patients (12.6%) were successfully pregnant during the postoperative follow-up. There were 44 recurrences (12.9%) and 6 deaths (1.8%). Lymphadenectomy had no significant influence on overall survival of patients with MOGCT (*P*=0.621; **Figure 1**) or disease-free survival rate (*P*=0.332; **Figure 2**). The OS rates at 5 years in the LND group and non-LND group were 99.3% vs. 100%, respectively. The DFS rates at 5 years in the LND group and non-LND group were 88.8% vs. 88.3%, respectively.

As shown in **Table 1**, before propensity score matching, there were significant differences in age, tumor size, stage, omentectomy, and hysterectomy rate between the two groups (*P* < 0.05). Patients in the non-LND group were younger, their tumor sizes smaller, and the rates of omentectomy and hysterectomy lower than in the LND group. The LND group contained more stage III patients. After propensity score matching, there were no significant differences between the LND and non-LND groups, indicating that the influence of the potential confounders was significantly decreased or eliminated by the algorithm. However, bleeding, operative time, and complications between the two groups exhibited statistically significant differences. Bleeding, operation time, and complications in the LND group were greater than those in the non LND group (*P*<0.05).

**Table 1:** Demographic and clinical characteristics of MOGCT patients before and after propensity score matching.

Parameter	Total(n=340)	Before Propensity Score Matching			After Propensity Score Matching		
		LND(n=143)	non-LND(n=197)	*P*	LND(n=102)	non-LND(n=102)	*P*
Age Mean ± SD	25.3±11.2	28.62±8.383	22.9±12.298	<0.001	27.38±7.454	27.89±11.147	0.701
Tumor size Mean ± SD	12.7±6.3	14.13±7.120	11.59±5.430	<0.001	13.25±6.804	13.37±5.646	0.892
Histopathology				0.158			0.760
IMT, immature teratoma	128(37.6)	45(31.5)	83(42.1)		34(33.3)	36(35.3)	
DSG, dysgerminoma	64(18.8)	23(18.2)	38(19.3)		21(20.6)	24(23.5)	
YST, yolk sac tumor	109(32.1)	53(37.1)	56(28.4)		39(38.2)	32(31.4)	
Mixed germ cell tumor	39(11.5)	19(13.3)	20(10.2)		8(7.9)	10(9.8)	
FIGO Stage				0.003			0.129
1	175(51.5)	74(51.7)	101(51.3)		53(52.0)	57(55.9)	
2	55(16.2)	21(14.7)	34(17.3)		16(15.7)	16(15.7)	
3	82(24.1)	44(30.8)	38(19.3)		31(30.4)	21(20.6)	
4	28(8.2)	4(2.8)	24(12.2)		2(2.0)	8(7.8)	
Surgery route				0.145			0.873
Laparoscopy	95(27.9)	34(23.8)	61(31.0)		27(26.5)	26(25.5)	
Laparotomy	245(72.1)	109(76.2)	136(69.0)		75(73.5)	76(74.5)	
Lymph node metastasis							
YES	18(5.3)	18(12.6)	–	–	13(12.7)	–	–
NO	322(94.7)	125(87.4)	–		89(87.3)	–	
Omentectomy				<0.001			0.773
YES	119(35.0)	70(49.0)	49(24.9)		38(37.3)	40(39.2)	
NO	221(65.0)	73(51.0)	148(75.1)		64(62.7)	62(60.8)	
Hysterectomy				<0.001			0.401
YES	40(11.8)	28(19.6)	12(6.1)		15(14.7)	11(10.8)	
NO	300(88.2)	115(80.4)	185(93.9)		87(85.3)	91(89.2)	
Blood loss (ml)	294.44±185.68	399.58±218.64	218.12±105.26	<0.001	398.33±218.12	230.20±124.30	<0.001
Operation time (min)	196.88±92.71	233.28±78.53	170.46±93.42	<0.001	240.46±84.29	158.24±79.21	<0.001
Complication				<0.001			<0.001
YES	55(16.2)	41(28.7)	14(7.1)		27(26.5)	5(4.9)	
NO	285(83.8)	102(71.3)	183(92.9)		75(73.5)	97(95.1)	
Adjuvant chemotherapy				0.638			0.470
YES	311(91.5)	132(92.3)	179(90.9)		94(92.2)	91(89.2)	
NO	29(8.5)	11(7.7)	18(9.1)		8(7.8)	11(10.8)	
Pregnancy rate(%)				0.196			0.548
YES	43(12.6)	22(15.4)	21(10.7)		16(15.7)	13(12.7)	
NO	297(87.4)	121(84.6)	176(89.3)		86(84.3)	89(87.3)	
Recurrence (%)				0.009			0.385
YES	44(12.9)	26(18.2)	18(9.1)		14(13.7)	10(9.8)	
NO	296(87.1)	117(81.8)	179(90.9)		88(86.3)	92(90.2)	
Mortality(%)				0.699			1.000
YES	6(1.8)	3(2.1)	3(1.5)		1(1.0)	2(2.0)	
NO	334(98.2)	140(97.9)	194(98.5)		101(99.0)	100(98.0)	

**Figure 3:**
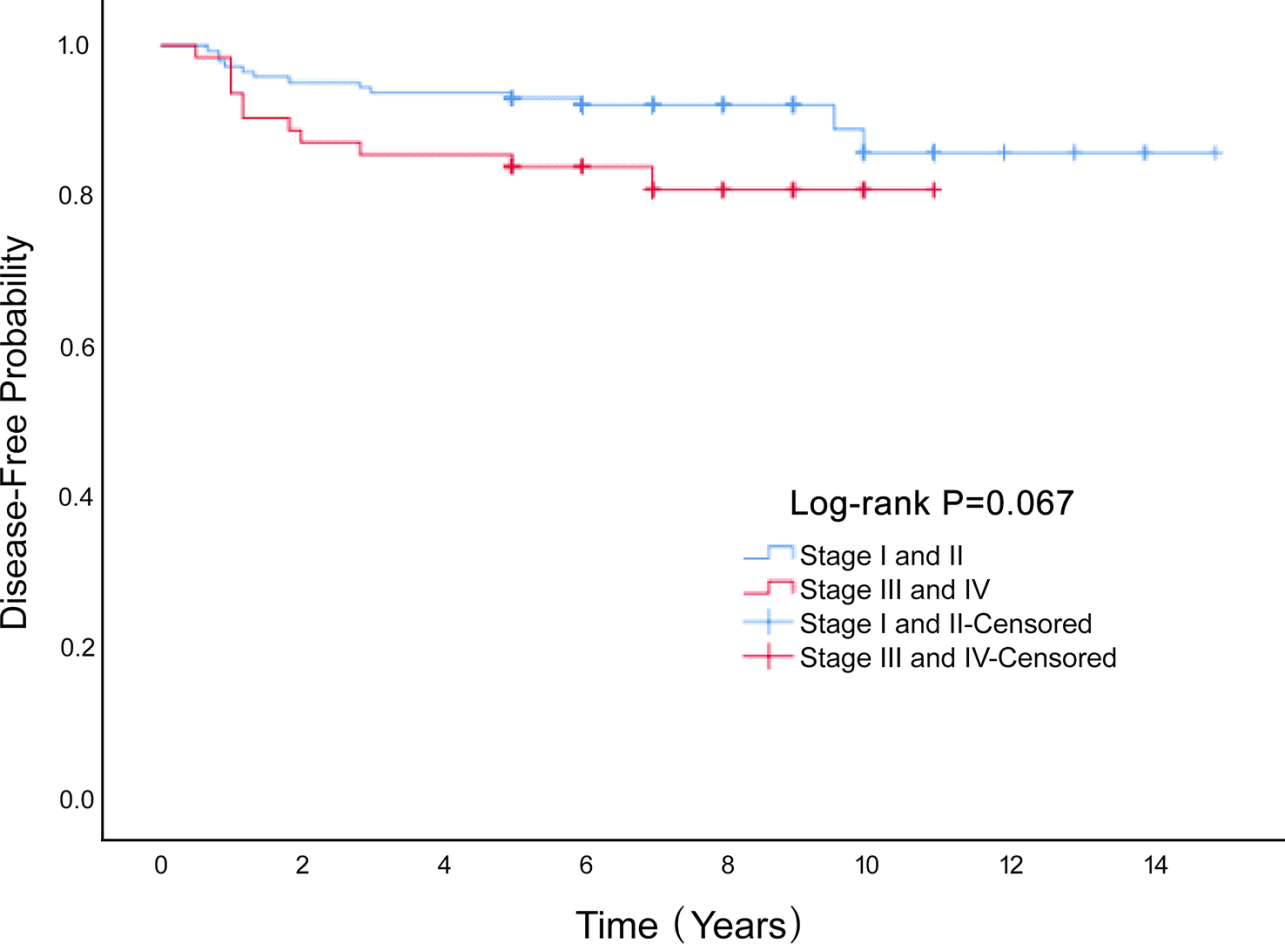
The Disease-Free survival rate of the Stage I and II and Stage III and IV groups of MOGCT patients.

Univariate Cox proportional hazard analyses were conducted on all clinical factors to explore their effect on disease-free survival based on propensity score matching (**Table 2**). Age (HR=1.040, 95%CI 1.007-1.073, *P*=0.016), stage (HR=2.084, 95%CI 0.933-4.656, *P*=0.073), laparoscopy (HR=2.416, 95% CI 0.902-6.472, *P*=0.079) and omentectomy (HR=0.414, 95% CI 0.155-1.109, *P*=0.079) was the factors affecting DFS. A multivariate Cox proportional hazard model was constructed on all the clinical characteristics with a prognostic value from the univariate analysis, and the results showed that advanced stage increased the rate of recurrence (HR=2.354, 95%CI 1.025-5.407, *P*=0.044). Stage had a significant influence on the disease-free survival rate of MOGCT (*P*=0.067) (**Figure 3**). The DFS rates at 5 years in the stage I/II, and stage III/IV were 90.8% vs. 84.5%, respectively.

**Table 2:** Cox-regression analysis of the factors for DFS on propensity score matching.

parameter	DFS							
	Univariate analysis				Multivariate logistic analysis			
	HR	95%CI		*P*	HR	95%CI		*P*
Age	1.040	1.007	1.073	0.016	1.030	0.998	1.603	0.069
Tumor size	0.961	0.894	1.034	0.289	NA	NA	NA	0.575
Histopathology:IMT、DSG、YST	Ref				Ref			
Histopathology:Mix-GCT	1.435	0.428	4.813	0.558	NA	NA	NA	NA
StageI、II	Ref				Ref			
StageIII、IV	2.084	0.933	4.656	0.073	2.354	1.025	5.407	0.044
Laparotomy	Ref				Ref			
Laparoscopy	2.416	0.902	6.472	0.079	1.593	0.683	3.713	0.281
non-Lymphadenectomy	Ref				Ref			
Lymphadenectomy	1.49	0.661	3.358	0.336	NA	NA	NA	NA
non-Omentectomy	Ref				Ref			
Omentectomy	0.414	0.155	1.109	0.079	0.411	0.148	1.142	0.088
non-Hysterectomy	Ref				Ref			
Hysterectomy	1.031	0.307	3.460	0.960	NA	NA	NA	NA
non-Chemotherapy	Ref				Ref			
Chemotherapy	2.699	0.363	20.050	0.332	NA	NA	NA	NA

**Figure 4:**
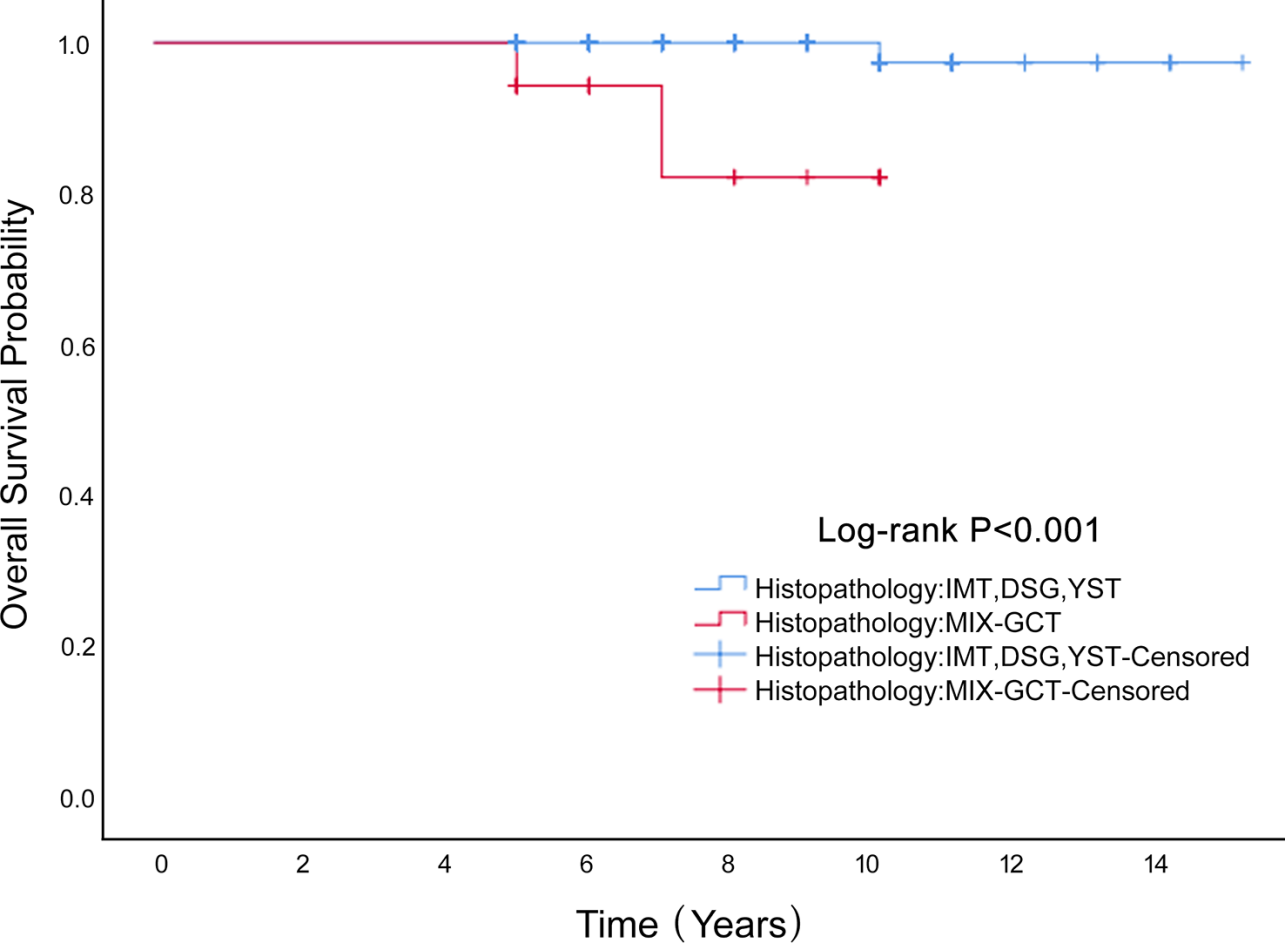
The Overall Survival rate of histopathology of MOGCT patients.

Univariate Cox proportional hazard analyses were conducted on all clinical factors to explore their effect on the overall survival based on propensity score matching (**Table 3**). The factors affecting OS were histopathology (HR=21.486, 95%CI 1.943-237.556 *P*=0.012), stage (HR=4.915, 95%0.444-54.429 *P*=0.194) and age (HR=1.084, 95%CI 1.011-1.162 *P*=0.024). After multivariate Cox regression analysis, the factors influencing age were not statistically significant *(P*=0.171). Mixed germ cell tumors had a worse outcome (HR=15.166, 95%CI 1.060-217.060 *P*=0.045). Mixed germ cell tumors were worse than the other three pathological types (*P <*0.001; **Figure 4**). In the six patients who died, four had a histological type of mixed germ cell tumor and two had yolk sac tumors. The OS rates at 5 years in the histopathology (IMT、DSG、YST) group and mixed germ cell tumor were 100% vs. 97.4%, respectively.

**Table 3:** Cox-regression analysis of the factors for OS on propensity score matching.

parameter	OS							
	Univariate analysis				Multivariate logistic analysis			
	HR	95%CI		*P*	HR	95%CI		*P*
Age	1.084	1.011	1.162	0.024	1.043	0.982	1.108	0.171
Tumor size	1.055	0.884	1.259	0.551	NA	NA	NA	NA
Histopathology:IMT、DSG、YST	Ref				Ref			
Histopathology:Mix-GCT	21.486	1.943	237.556	0.012	15.166	1.060	217.060	0.045
StageI、II	Ref				Ref			
StageIII、IV	4.915	0.444	54.429	0.194	1.773	0.109	28.793	0.687
Laparotomy	Ref				Ref			
Laparoscopy	0.032	0.000	2644.406	0.552	NA	NA	NA	NA
non-Lymphadenectomy	Ref				Ref			
Lymphadenectomy	0.551	0.050	6.079	0.626	NA	NA	NA	NA
non-Omentectomy	Ref				Ref			
Omentectomy	0.906	0.082	10.023	0.936	NA	NA	NA	NA
non-Hysterectomy	Ref				Ref			
Hysterectomy	3.778	0.340	42.004	0.279	NA	NA	NA	NA
non-Chemotherapy	Ref				Ref			
Chemotherapy	25.416	0.000	73474732.290	0.670	NA	NA	NA	NA

## 4 Discussion

There is controversy regarding the current guidelines on performing lymphadenectomy. The European Society of Gynecological Oncology (ESGO) and European Society for Medical Oncology (ESMO) recommend that lymphadenectomy be performed only if there is evidence of lymph node abnormalities in MOGCTs (8, 9). The National Comprehensive Cancer Network (NCCN) recommends comprehensive staging surgery with or without fertility sparing for patients with MOGCT (10). Our study analyzed the prognostic value of lymphadenectomy in 340 patients with MOGCTs. Survival, univariate, and multivariate Cox proportional analyses showed no prognostic differences between the LND and non-LND groups.

Our study showed that lymphadenectomy did not affect the prognosis of MOGCT, regardless of stage. There was also no difference in recurrence rate. Several prior studies have explored lymphadenectomy in MOGCTs and reported that LND is not an independent prognostic factor. Particularly, Nasioudis et al. compared 1287 patients with MOGCT who underwent LND and 1210 who did not undergo LND and concluded that LND did not correlate with OS in apparent early stage MOGCTs (3). Mahdi et al. included 493 590 early stage MOGCT patients with and without LND, respectively, and reported that LND was not associated with survival benefit in early stage MOGCT (11). Wang et al. and Chen et al. reported that lymphadenectomy had little impact on survival in stages I and II, but increased survival in stages III and IV (12, 13). This may be because the node metastasis is lower in early than advanced stage ovarian cancer. Some authors thought retroperitoneal lymphadenectomy provided no survival advantages in either early or late stage ovarian cancer, but was associated with surgical complications, although it did help avoid adjuvant chemotherapy in early stage (14). Thus, only enlarged lymph nodes were recommended to resected to achieve complete cytoreduction which was associated with good oncological outcome (14, 15).

Our study found that stage was an independent prognostic factor for DFS in the multivariate analysis, but not for OS. We explored the optimal outcomes because of the common application postoperative chemotherapy in our study based on the NCCN guidelines. After the introduction of chemotherapy, the survival rate of MOGCT drastically improved. A prior study reported that after administration of three cycles of chemotherapy with bleomycin, etoposide, and cisplatin (BEP), the sustained remission rate had exceeded 95% (11). Mangili et al. reported that incomplete surgical staging can increase the recurrence but not the survival rate in MOGCTs, because salvage chemotherapy at the time of relapse can result in excellent outcomes (16). Additionally, histopathology was an independent prognostic factor associated with OS in the multivariate analysis in our study. Previous studies obtained contrasting results regarding histopathology and prognosis in the patients with MOGCT. For example, Chan et al. reported histopathology was an independent prognostic factor for improved survival multivariate analysis (7). However, Chen et al. revealed that among different types of MOGCT, histopathology was not associated with different survival rates (12).

Univariate Cox proportional hazard analyses showed age was a prognostic factor for both DFS and OS. Accumulating evidence suggests that the management of ovarian cancer should be personalized considering the performance status of the patient, especially in older patients. Preoperative frailty assessment is important for predicting surgical complications and determining personalized treatment (17). A systematic review reported that frail patients are more prone to experience 30-day postoperative complications, non-home discharge, ICU admission, and worse oncologic outcomes (18). Thus, the decision on whether to perform lymphadenectomy should take age into account. Several studies recommend that lymphadenectomy not be performed in young women and pediatric patients with MOGCTs (11, 13).

The pregnancy rates in the LND and non-LND groups were 15.4% and 10.7%, respectively. Consistent with previous work, pregnancy rates in the LND and non-LND groups were not statistically different after propensity score matching (19).

Our study added more data to the current studies on the effect of lymphadenectomy in MOGCTs. The main limitation of this study was that its retrospective nature, which also restricted its follow-up strength. Furthermore, the low incidence of this disease has hampered the use of randomized controlled trials.

## Data availability statement

The original contributions presented in the study are included in the article/supplementary material. Further inquiries can be directed to the corresponding authors.

## Ethics statement

The studies involving human participants were reviewed and approved by the Ethics Committee of the West China Second University Hospital. Written informed consent to participate in this study was provided by the participants’ legal guardian/next of kin.

## Author contributions

Data curation was contributed by LH and BL. LH, AZ and BL were responsible for the conception of the paper and manuscript drafting. All authors contributed to the revision and final approval of the manuscript. All authors contributed to the article and approved the submitted version.
